# The Effect of Pretreatment on a PtCu/C Catalyst’s Structure and Functional Characteristics

**DOI:** 10.3390/ijms24032177

**Published:** 2023-01-22

**Authors:** Sergey Belenov, Alina Nevelskaya, Alexey Nikulin, Mikhail Tolstunov

**Affiliations:** 1 Chemistry Faculty, Southern Federal University, 7 Zorge St, 344090 Rostov-on-Don, Russia; 2 Prometheus R&D LLC, 4g/36 Zhmaylova St, 344091 Rostov-on-Don, Russia; 3 Federal State Budgetary Institution of Science “Federal Research Centre The Southern Scientific Centre of the Russian Academy of Sciences” (SSC RAS), St. Chehova, 41, 344006 Rostov-on-Don, Russia

**Keywords:** electrocatalyst, bimetallic nanoparticles, acid treatment, heat treatment, oxygen electroreduction reaction, stability

## Abstract

This research focuses on studying the effects of various pretreatment types on a PtCu/C catalyst synthesized by the co-deposition of metal precursors. The treatment in a 1 M HNO_3_ solution for 1 h is shown to result in a slight increase in activity in the oxygen electroreduction reaction (both the mass activity and specific activity calculated for the value of the electrochemically active surface area). The sample obtained after the thermal treatment, which is carried out at 350 °C under an argon atmosphere for 1 h, demonstrates 1.7 times higher specific activity than the sample before the treatment. The durability testing results obtained by the stress testing method in a potential range of 0.6–1.4 V during 2000 cycles show that the PtCu/C catalysts after both the acid treatment and the thermal treatment are characterized by higher residual activity than the sample in the “as-prepared” state.

## 1. Introduction

Alternative ways of producing energy are becoming increasingly popular in the modern world [1]. For example, fuel cells (FCs) play a vital role in the fields of transport, unmanned technologies, portable gadgets, etc. An essential component of FCs is the Pt-based catalyst [2,3,4,5,6,7,8]. To enhance the performance of this catalyst and to reduce its cost, the method of alloying platinum with different d-metals (Co, Cu, Ni, Cr, and Fe) is used [9,10]. These catalysts can be characterized by higher activity and durability while reducing the cost of the entire system.

At the same time, the application of bimetallic catalysts has its potential shortcomings, including the dissolution of the non-noble component from nanoparticles (NPs) of the catalyst. This process has a negative impact on the FCs’ operating time due to the displacement of protons by dissolved ions in the proton-conducting membrane, thus, leading to a decrease in its conductivity.

To prevent the dissolution process of the alloying component, various types of catalyst pretreatments are used, including thermal treatment and treatment in different acids. Each of these approaches, as well as their combination, allows varying the parameters of conducting the treatment, which in turn affects the structural and functional characteristics of the obtained catalysts [11,12].

The treatment of bi- and trimetallic catalysts by exposing them to acids is a promising and scalable method to obtain materials with a decreased content of alloying components [13,14,15,16,17,18,19,20]. This method can be used by varying the composition of acids (HNO_3_, H_2_SO_4_, HClO_4_, etc.), their concentration (from dilute acids to more concentrated ones), and the acid exposure time (from several hours to 24 h). These types of catalyst pretreatments can be conducive to the formation of improved “core–shell” structures of NPs due to the dissolution of the alloying component from the NPs’ surface, with only the platinum shell left [21]. As described in the reference [14], the formation of hollow bimetallic NPs characterized by high mass activity in the oxygen electroreduction reaction (ORR) is also possible. Nevertheless, in some instances, in the case of acid treatments of catalysts with a core–shell structure and in the presence of NPs with defects in their platinum shell in the system, the dissolution of the d-metal core results in a decrease in the durability of these catalysts due to the almost entire dissolution of the alloying component [13].

Thermal treatment is conducted in various gas media (Ar, N_2_, and H_2_), including their mixtures in different ratios [22]. Notably, in the case of bi- and trimetallic catalysts, thermal treatment can be conducive to the formation of the ordered NPs’ structure [21,23,24,25,26,27]. The formation of the intermetallic structure can, in turn, lead to an increase in the materials’ ORR activity and durability [21,23,24]. The undesirable effects resulting from thermal treatment include the growth of NPs’ size [27,28,29,30,31,32], which leads to a decrease in the electrochemically active surface area (ESA) [31,32,33,34,35,36,37]. However, even then, a decrease in the specific activity does not always occur. For example, as described in the references [21,24,27], an increase in both the mass ORR activity and the specific ORR activity calculated for the value of the ESA was observed after thermal treatment. Apart from the possible formation of the ordered solid solution structure, an increase in the size of NPs is also conducive to the growth of the catalysts’ durability. Nevertheless, thermal treatment does not always result in the enhancement of the catalysts’ functional characteristics [38,39]. For example, the reference [38] describes the presence of an ORR activity peak at 300 °C, although any further heating of the samples leads to no positive changes in their structure and functional characteristics. Therefore, the question of varying the conditions of conducting thermal treatment (temperature, atmosphere, and duration) for different Pt-based catalysts is still a worthy subject of research.

This work is a continuation of a previously conducted study [40]. This research is aimed at studying the effects of thermal treatment and treatment in acids, including different combinations of these pretreatment types, on a PtCu/C catalysts’ microstructure and electrochemical behavior.

## 2. Results

According to the results of total reflection X-ray fluorescence (TXRF) analysis, the Pt:Cu ratio in the obtained PtCu/C materials is 1:1.26 for the samples in the as-prepared state and after the thermal treatment (Table 1). The Pt:Cu ratio is 1:0.29 for the materials obtained after the acid treatment as well as after the treatment in acids followed by the thermal treatment. The Pt:Cu ratio is 1:0.80 for the material obtained after the thermal treatment with the successive acid treatment. Therefore, the treatment in dilute nitric acid results in a substantial dissolution of copper for all the PtCu/C materials studied. At the same time, the thermal treatment leads to a 63% decrease in the proportion of copper dissolved during the acid treatment compared with the heat-untreated material.

**Figure 1:**
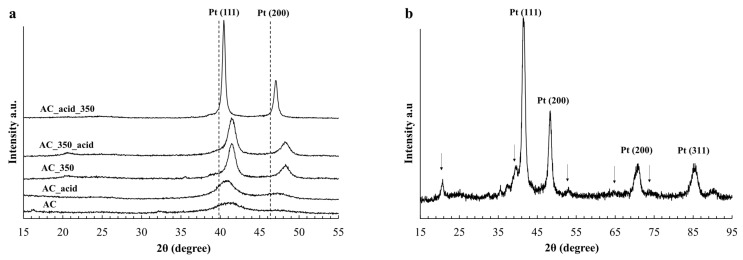
The X-ray diffraction patterns of the obtained PtCu/C samples (**a**); the X-ray diffraction pattern of the AC_350 sample (**b**) indicating the localization of the PtCu intermetallic structure.

According to the results of X-ray powder diffraction (XRD) analysis of the obtained PtCu/C catalysts, the samples were established to contain a carbon phase and a phase of platinum with a face-centered cubic structure. In this regard, the maxima of the platinum reflection are shifted to a high-angle region of 2θ relative to the localization of the platinum-phase reflection maxima that are equal to 39.8° and 46.3° 2θ (Figure 1a). This indicates the formation of the PtCu solid solution. The crystal lattice parameter for the PtCu solid solutions was calculated with regard to the localization of reflections (Table 1).

The composition of the solid solution was estimated according to Vegard’s law [41]. The X-ray diffraction patterns of the AC_350 sample obtained after the thermal treatment under an inert atmosphere at 350 °C demonstrate a shift in the reflection maxima (111) and (200) to the high-angle region of 2θ relative to the sample in the as-prepared state, which indicates the complete alloying of Pt and Cu after the thermal treatment. Nevertheless, the reflection maxima (111) and (200) of the AC_acid sample obtained after the acid treatment shifted to the low-angle region of 2θ compared with the untreated sample (Table 1). It is noteworthy that the composition determined by TXRF analysis and the solid solution composition calculated according to Vegard’s law on the basis of the XRD data for the obtained AC sample do not coincide, which may be caused by the incomplete penetration of copper atoms into the solid solution with platinum. Despite the absence of any additional reflections corresponding to copper oxides in the X-ray diffraction patterns (Figure 1), the sample may contain X-ray amorphous copper oxide formed by copper atoms not included in the composition of the PtCu solid solution. This assumption is indirectly supported by the composition of the material obtained after the acid treatment that contains less copper (PtCu_0.29_) compared with the solid solution composition of the acid-untreated AC material. It is assumed that the acid treatment results in the complete dissolution of copper not included in the solid solution composition and the partial dissolution of copper from the surface of the bimetallic NPs of the PtCu solid solution [21]. The complete alloying of the material after the thermal treatment, with the solid solution composition changing from PtCu_0.56_ to PtCu_0.88_, leads to a significant decrease in the proportion of copper dissolved after the acid treatment. For example, the AC_350 material obtained after the thermal treatment changes its composition to PtCu_0.80_ during the acid treatment in contrast to the untreated AC material that degrades to PtCu_0.29_ with much less copper in the composition.

The average crystallite size calculated by the reflex width at half maximum for the obtained materials shows that the acid treatment results in virtually no changes in the average crystallite size (Table 1). The thermal treatment leads to the enlargement of crystallites from 2.9 to 7.8 nm for the AC_350 sample and to 20.2 nm for the AC_acid_350 sample obtained after the acid treatment followed by the thermal treatment. This phenomenon is caused by a decrease in the proportion of copper in the sample, which is conducive to the substantial enlargement of NPs during the subsequent thermal treatment [40].

According to the results of XRD analysis, the AC_350 sample obtained after the thermal treatment is characterized by the formation of a PtCu intermetallic compound with a hongshiite structure (the R3m space group) [35], with the reflection maxima roughly localized at 21°, 39°, 53°, 64°, and 74° 2θ (Figure 1b).

**Figure 2:**
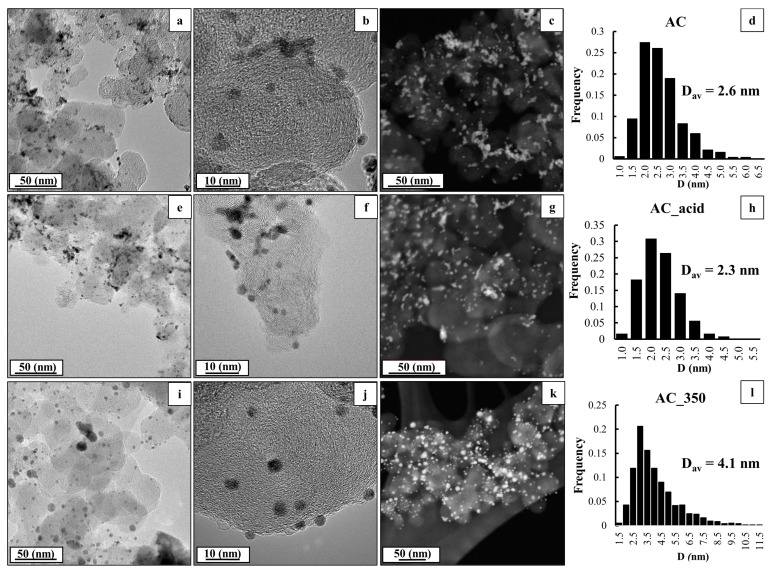
The TEM micrographs of the PtCu/C samples (**a**,**b**,**e**,**f**,**i**,**j**); the STEM micrographs (**c**,**g**,**k**); and the histograms of the NPs’ size distribution (**d**,**h**,**l**) before and after the treatment.

The TEM and STEM results for the AC, AC_acid, and AC_350 materials (Figure 2) exhibit a uniform distribution of NPs over the carbon support surface with a small proportion of agglomerates. The obtained AC sample is characterized by a narrow size dispersion (Figure 2d) and an average size of NPs equal to 2.6 nm, which correlates well with the average crystallite size based on the XRD data (Table 1). The acid treatment results in virtually no changes in the average size of NPs and the nature of their size distribution (within the margin of error) (Figure 2h), which is compliant with the XRD data. At the same time, the thermal treatment results in a significant increase in the average size of NPs from 2.6 to 4.1 nm according to the TEM results. This enlargement is accompanied by a change in the nature of the NPs’ size distribution due to an increase in the proportion of larger particles and a decrease in the proportion of smaller NPs (Figure 2l). This fact is compliant with the enlargement of the crystallite size based on the XRD data, although the size of particles according to the TEM results proved to be smaller than the average crystallite size determined with regard to the XRD results. This appears to be connected with the features of determining the average size of NPs by TEM due to the presence of agglomerates, the contribution of which was not taken into account [40].

**Figure 3:**
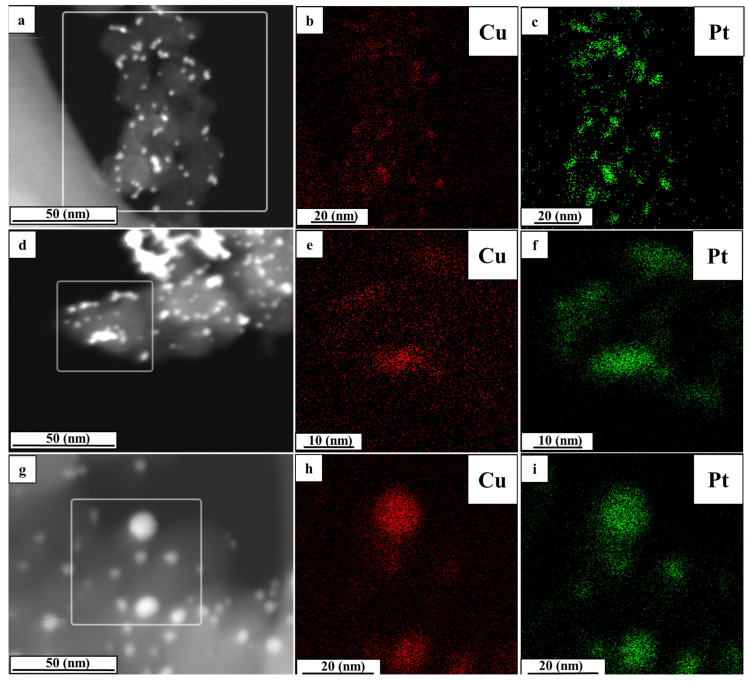
The elemental mapping of the AC (**a**–**c**), AC_acid (**d**–**f**), and AC_350 (**g**–**i**) samples.

According to the results of the elemental mapping (Figure 3), atoms of copper and platinum are localized at the same sections, which confirms the formation of bimetallic PtCu NPs. The samples’ local composition, determined based on the results of the elemental mapping, as well as their total composition, determined by TXRF analysis, coincide with the material in the as-prepared state (PtCu_1.26_). However, there are some variances for the materials obtained after the thermal treatment and acid treatment. The proportion of copper in individual NPs increases for the AC_350 sample (the obtained composition is PtCu_1.86_), which is not compliant with the TXRF results (the obtained composition is PtCu_1.26_). According to the TEM results, the content of copper decreases after the acid treatment (the obtained composition is PtCu_0.46_) but to a lesser extent than as revealed by TXRF analysis (the obtained composition is PtCu_0.29_). Therefore, the results of the elemental analysis and the local microanalysis by TEM demonstrate the same trends of change in the composition. However, in some instances, they differ in absolute values due to the features of the methods used.

**Figure 4:**
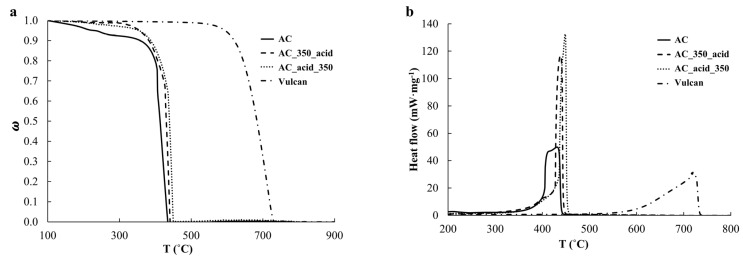
The TGA (**a**) and DSC (**b**) curves of the AC, AC_350_acid, and AC_acid_350 catalysts.

To study the features of the high-temperature oxidation of the obtained AC, AC_350_acid, and AC_acid_350 materials, TGA was conducted. The intense oxidation of the AC sample in the as-prepared state is revealed to proceed at lower temperatures compared with the AC_350_acid and AC_acid_350 samples and the Vulcan carbon support (Figure 4a). The nature of the combustion of these samples also differs. For example, the untreated material burns in a wider range of temperatures than the materials obtained after various pretreatment types (Figure 4b) [42,43]. The combustion of the Vulcan carbon support occurs at higher temperatures and a wider range of temperatures compared with the PtCu/C catalysts.

After studying the materials’ composition and structure, their electrochemical characteristics were estimated in the three-electrode cell. To standardize the electrodes, the method of the repeated (100 cycles of the CV) potential sweep in the studied potential range was used. The obtained CVs (Figure S1) have an appearance typical for Pt-based catalysts. The CVs exhibit hydrogen, oxygen, and double-layered regions. Figure S1 demonstrates that for surface standardization, the AC and AC_acid catalysts require more cycles (80 and 70 cycles, respectively) than the AC_350 sample (40 cycles) (Figure S1a). It is worth noting that the CV of the AC_350 catalyst obtained after the thermal treatment exhibits an anodic peak at ~0.75 V that decreases its intensity after 10–15 cycles (Figure S1c). This conforms to the dissolution of copper from the solid solution phase [38,39,40]. Moreover, the peak of the dissolution of copper from its own phase was registered at ~0.35 V during the first cycle of the CV for the AC_350 material. The data obtained testify to the change in the structure of NPs after the thermal treatment, as a result of which due to the interdiffusion of atoms of copper and platinum, copper atoms migrate from the volume to the surface of NPs and undergo dissolution during standardization. Moreover, the total amount of copper dissolved during the standardization was determined. It is equal to 7.7% of the total amount of copper in the material.

The study of the composition of NPs for the catalysts before and after the electrochemical measurements using TXRF analysis revealed that after 100 cycles of the CV, the AC_350 material obtained after the thermal treatment is characterized by the least amount of dissolved copper (the change in the composition from PtCu_1.26_ to PtCu_0.89_) compared with the untreated AC sample (PtCu_1.26_ → PtCu_0.25_). The AC_acid material obtained after the acid treatment is characterized by the least amount of copper left in the composition of NPs (PtCu_0.29_ → PtCu_0.21_). This phenomenon may be caused by the chemical dissolution of the X-ray amorphous copper oxide in the acidic medium, which is not recorded in the CVs.

**Figure 5:**
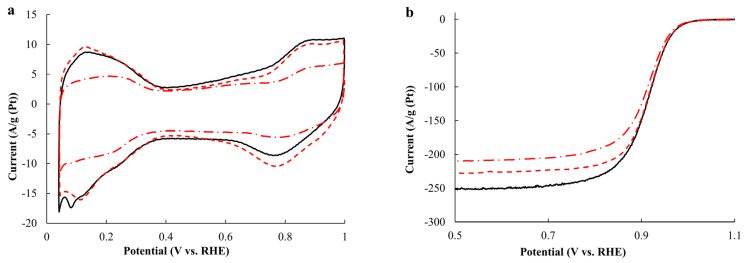
The cyclic voltammograms (**a**) and the linear sweep voltammograms at 1600 rpm (**b**) of the AC (the solid line), AC_acid (the dashed line), and AC_350 (the dash-dotted line) samples; the electrolyte is HClO_4_; and the potential sweep rate is 20 mV/s.

The ESA value calculated according to the adsorption/desorption of atomic hydrogen (Figure 5a) after the standardization for the sample in the as-prepared state decreases from 37 m^2^/g_Pt_ to 20 m^2^/g_Pt_ after the thermal treatment. The charge amount consumed for the adsorption/desorption of hydrogen differs slightly due to the hydrogen spillover effect, i.e., the chemical migration of hydrogen from the metal surface after its adsorption [44,45]. Therefore, the charge amount consumed for the adsorption of atomic hydrogen should exceed the charge amount consumed for its desorption. The ESA has been calculated with regard to both the charge amounts by the average value. Notably, the ESA value does not practically change for the AC_acid material obtained after the acid treatment (Table 2). The ESA value for the AC_350_acid sample obtained after the thermal treatment followed by the acid treatment is slightly above the ESA value for the AC_350 sample obtained after the thermal treatment, which appears to be due to defects on the surface of NPs after the dissolution of copper. The highest decrease in the ESA value is registered for the AC_acid_350 material (16 m^2^/g_Pt_), which correlates well with the XRD results, where the size of crystallites for this catalyst is the biggest among the other PtCu/C samples studied (Table 1). It should be noted that the ESA value for the commercial JM40 catalyst exceeds all the ESA values for the PtCu/C materials presented.

The value of mass activity in the ORR calculated from the LSVs recorded at different rotation speeds in the RDE (Figure 5b) at a potential of 0.9 V is 2.1 times higher for the obtained AC material than for the commercial Pt/C catalyst. The acid treatment of the PtCu/C catalyst slightly increases its activity to 351 A/g, which is the highest activity per platinum mass among the studied samples (Table 2). A slight decrease in the mass activity of the sample obtained after the thermal treatment (from 332 to 308 A/g_Pt_) can also be observed. At the same time, the specific activity calculated for the ESA value increases by 1.7 times, which is the highest value of specific activity among the studied catalysts (Table 2). A comparison of the half-wave potential values for the studied catalysts has shown that the AC_acid material is characterized by the highest half-wave potential, whereas the AC_acid_350 sample obtained after the acid treatment followed by the thermal treatment exhibits the lowest value as well as the lowest value of specific activity (Table 2). The number of electrons calculated according to the Koutetsky−Levich equation for the PtCu/C materials varies in the range of 3.6–3.9 electrons, which is slightly below the number for the Pt/C catalyst (~4.0 electrons). This difference is due to the effect of the alloying component [18,38,46].

Therefore, the activity values calculated from specific currents at a potential of 0.9 V and from the half-wave potential are correlated. It is noteworthy that the commercial JM40 catalyst exhibits lower ORR activity (both the mass activity and specific activity per m^2^Pt) than the PtCu/C samples obtained (AC, AC_acid, and AC_350).

**Figure 6:**
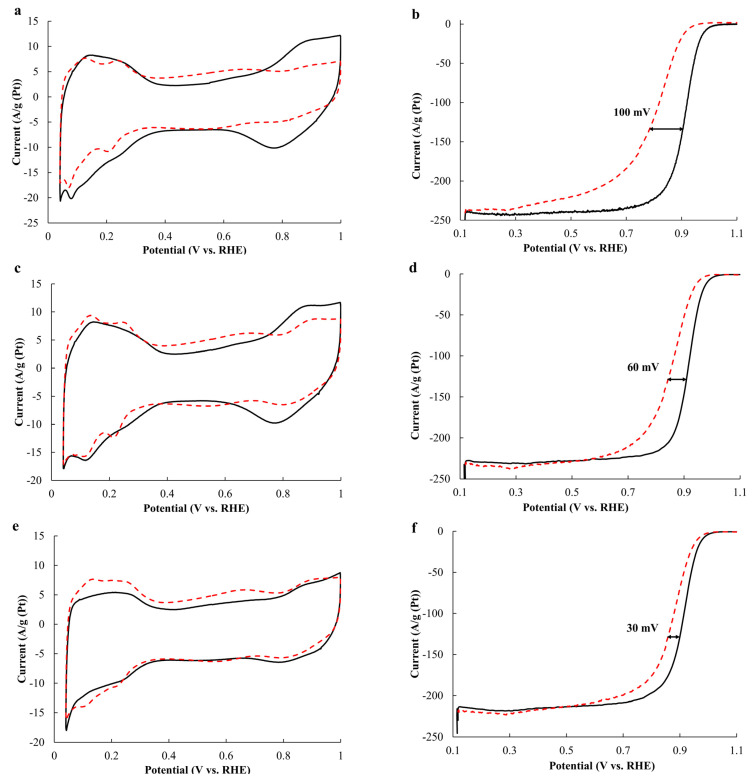

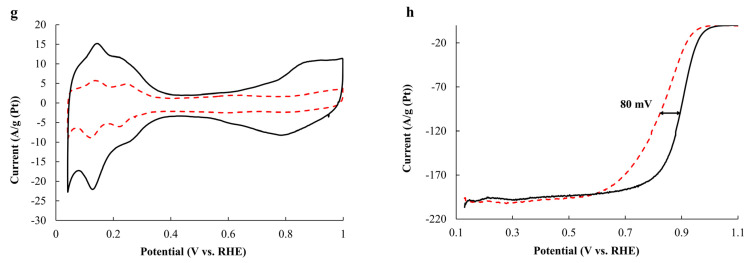
The cyclic voltammograms (**a**,**c**,**e**,**g**) and the linear sweep voltammograms (**b**,**d**,**f**,**h**) of the AC, AC_acid, and AC_350 samples and the JM40 catalyst before (the solid line) and after (the dashed line) 2000 cycles in a potential range of 0.6–1.4 V.

The half-wave potential for the AC catalyst calculated from the potentiodynamic curves under an oxygen atmosphere decreases to a greater extent (from 0.91 to 0.81 V) after 2000 cycles of the CV than for the AC_acid and AC_350 materials, for which the half-wave potential changes from 0.92 to 0.86 V and from 0.91 to 0.88 V, respectively (Figure 6b,d,f).

**Figure 7:**
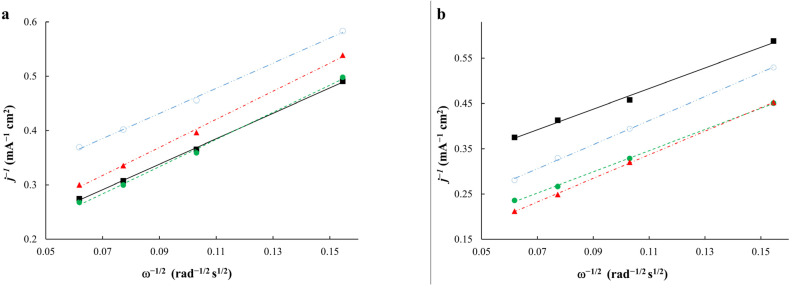

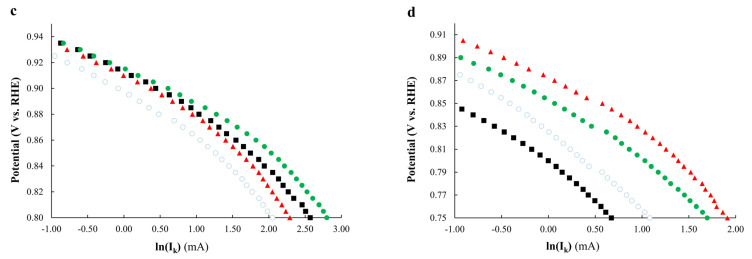
The Koutetsky−Levich dependence at a potential of 0.90 V before stress testing (**a**) and at a potential of 0.80 V after stress testing (**b**); the Tafel dependence before (**c**) and after (**d**) stress testing for the following materials: black−AC, green−AC_acid, red−AC_350, and blue−JM40.

The values of kinetic currents and the number of electrons of the cell reaction before and after stress testing were determined according to the Koutetsky−Levich equation (Figure 7a,b). It should be noted that the values of kinetic currents decrease significantly after stress testing due to the degradation processes. Taking into account degradation, the number of electrons (n) after durability testing was determined at 0.80 V. The analysis of the slope of the Koutetsky−Levich dependences (Figure 7a,b) before and after durability testing shows that the slope angle for different materials before and after stress testing is similar. The calculation of the number of electrons for the materials after stress testing also shows values of about 3.6–3.9, which is close to the four-electron mechanism of the reaction. The number of electrons of less than four may testify to the contribution of the concurrent two-electron reaction with the formation of hydrogen peroxide [47].

The results of the determination of the mass activity and specific activity calculated for the ESA value after stress testing show that the AC_350 catalyst obtained after the thermal treatment is characterized by the highest residual activity of 352 A/g_Pt_ and 15 A/m^2^_Pt_ at a potential of 0.85 V. The material in the as-prepared state, for which it is impossible to determine the activity of at a potential of 0.85 V after stress testing (Table 3), has the lowest residual activity among the PtCu/C samples. Notably, the commercial JM40 catalyst exhibits the lowest mass activity among all the materials presented (Table 3).

The analysis of the slope of the Tafel curves shows that the obtained materials (Figure 7c) are characterized by a slope of about 60 mV in a potential range of more than 0.85 V, which is typical for Pt-based catalysts [35,48]. It is worth noting that the dependences in the coordinates of E-ln(I_k_) demonstrate that the AC_acid material obtained after the acid treatment is the most active catalyst in the potential range of less than 0.90 V. Moreover, all the bimetallic systems are superior to the commercial Pt/C catalyst. The AC_350 sample exhibits the highest ORR activity after stress testing compared with the other materials studied. The results of stress testing show that the AC sample degraded to the greatest extent. At the same time, its activity proved to be lower than for the commercial Pt/C catalyst (Figure 7d). Therefore, in terms of their durability, the studied catalysts can be arranged in the following order: AC_350 > AC_acid > JM40 ≥ AC.

## 3. Materials and Methods

### 3.1. Synthesis and Pretreatment

The PtCu/C catalyst was synthesized by the coreduction of the platinum and copper precursors, as described in the reference [49]. The synthesis of the PtCu/C sample containing the bimetallic NPs with the alloy structure (the solid solution). The reduction was carried out using a 0.5 M sodium borohydride solution as the reducing agent taken in excess. The theoretical ratio of Pt:Cu was 1:2, with the platinum mass fraction equal to 30%.

The acid treatment of the material was conducted using a 1 M HNO_3_ solution for 1 h. The acid treatment (20 mL of 0.1 M HNO_3_ per 0.1 g of the catalyst) was performed at room temperature using a magnetic stirrer. The suspension was then filtered using the Büchner funnel with repeated rinsing with water and dried over P_2_O_5_. The material was marked as AC_acid.

The thermal treatment of the samples was conducted using the PTK-1.2-40 furnace (Teplopribor Research and Production Enterprise, Yekaterinburg, Russia) under an argon atmosphere at 350 °C, as described in the reference [40]. To refer to the heat-treated materials, the indices corresponding to the treatment temperature were added to the designation. The material was marked as AC_350. When combining both types of catalyst pretreatments, the samples were marked as AC_350_acid and AC_acid_350, indicating a pretreatment type sequentially.

### 3.2. Characterization

The Pt:Cu ratio in the PtCu/C samples was determined by total reflection X-ray fluorescence (TXRF) analysis using an RFS-001 spectrometer with the total external reflection of the X-ray radiation (Research Institute of Physics, Southern Federal University, Rostov-on-Don, Russia). The spectrum acquisition time was 300 s. The obtained material with a composition of PtCu_1.26_ was marked as AC. The metals’ mass fraction in the samples was determined from the mass of the unburned residue left after the calcination of the samples at 800 °C and presumably consisting of Pt and CuO.

The X-ray diffraction patterns of the PtCu/C materials were recorded using an ARL X’TRA powder diffractometer (CuKα) with a 2θ angle range of 15–95°, a detector movement step of 0.04°, and a scanning rate of 2° per minute. The average crystallite size was determined by the Scherrer formula [50]: D = Kλ/(FWHM·cosθ), where K = 0.98 is the Scherrer’s constant, λ is the wavelength of the monochromatic radiation (in Å), FWHM is the reflex full width at half maximum (in radians), D is the average crystallite size (nm), and θ is the angle of incidence (in radians).

The analysis of the samples by the transmission electron microscopy (TEM) and scanning transmission electron microscopy (STEM) methods was conducted using an FEI Tecnai G2 F20 S-TWIN TMP microscope (FEI, Hillsboro, OR, USA) with an EDAX EDS attachment operating at an accelerating voltage of 200 kV. The powders of the electrocatalyst were dispersed in ethanol for 2–3 min. Next, a drop of the suspension was applied to the copper grid covered with a thin layer of amorphous carbon. The number of processed NPs for each catalyst was 720–1150.

The thermal analysis of the obtained materials was conducted using the simultaneous thermal analyzer, NETZSCH STA 449 C Jupiter (Erich NETZSCH GmbH & Co. Holding KG, Selb, Germany) for thermal gravimetric analysis (TGA) and differential scanning calorimetry (DSC) under an atmosphere consisting of N_2_ (80%) and O_2_ (20%) in a temperature range of 25–800 °C at a heating rate of 10 °C/min and a gas flow rate of 20 mL/min with the use of alumina crucibles. The thermograms are given in dimensionless units (ordinate), where the mass fraction of the reacted carbon (ω) [42] was determined according to the formula: ω = (m_t_–m_t = 800_)/(m_t = 120_–m_t = 800_), where mt is the weight of the sample at a given temperature, m_t = 800_ is the weight of the sample at 800 °C, and m_t = 120_ is the weight of the sample at 120 °C.

### 3.3. Electrocatalytic Test

The electrochemical measurements were conducted in a three-electrode cell using 0.1 M HClO_4_ as the electrolyte. The electrolyte was preliminarily purged with Ar for 30 min. To study the catalysts, catalytic “inks” were used. The procedure of their preparation and deposition is described in the reference [40]. When preparing the inks, a weighed amount (6 mg) of catalyst was added to the mixture containing 1800 µL of isopropanol, 100 µL of deionized water, and 100 µL of the 1% aqueous emulsion of the Nafion^®^ polymer. Next, the suspension was stirred using a magnetic stirrer for 5 min and dispersed in the ultrasonic bath for 10 min. A 3 μL aliquot was applied to a rotating disk electrode using a micropipette and dried during the rotation at a speed of 700 rpm. When the catalytic layer was dried, the next 3 μL aliquot was applied and dried to form a uniform catalytic layer. The changes were made to the text of the article.

The standardization of the catalyst’s surface is an essential stage of electrochemical measurements. A total of 100 cycles of cyclic voltammetry (CV) in a potential range of 0.04–1.0 V at the rate of a potential sweep of 200 mV/s were recorded in a 0.1 M HClO_4_ solution saturated with Ar. Next, two cyclic voltammograms (CVs) were recorded in the same potential range at the rate of a potential sweep of 20 mV/s to determine the ESA value. When calculating the ESA value, the charge amount consumed for the adsorption/desorption of atomic hydrogen was taken into account. All the potentials given in this work are determined with regard to the reversible hydrogen electrode (RHE).

To determine the ORR activity of the catalysts, the linear sweep voltammograms (LSVs) at several disk electrode rotation speeds (400, 900, 1600, and 2500 rpm) were recorded utilizing the rotating disk electrode (RDE) technique. The electrolyte was a 0.1 M HClO_4_ solution saturated with O_2_ (1 h). The calculation of the kinetic current was conducted using the Koutetsky−Levich equation: 1/j = 1/j_k_ + 1/j_d_, where j is the experimentally measured current, jk is the kinetic current, and jd is the diffusion current (at a potential of 0.90 V and with regard to the RHE). Next, using the kinetic current value, the mass activity (A/g_Pt_) and specific activity calculated for the ESA value (A/m^2^_Pt_) were determined. The LSVs were also corrected by subtracting the equivalent curve, which was obtained on the same electrode under an Ar atmosphere, from the voltammogram: I (O_2_)–I (Ar), as described in the reference [51].

All the curves were preliminary normalized to take into account the contribution of the ohmic voltage drop according to the formula: E = E_set_–I·R, where E_set_ is the set value of the potential, and I·R is the ohmic potential drop equal to the product of the current strength and the resistance of the solution layer between the reference electrode and the studied one.

The durability of the catalysts was determined by the stress testing method, i.e., repeated (2000 cycles of the CV) cycling at the rate of a potential sweep of 100 mV/s in a potential range of 0.6–1.4 V in a 0.1 M HClO_4_ solution saturated with Ar, as described in the reference [40].

## 4. Conclusions

A systematic study of the effects of various pretreatment types and their combinations on the activity and durability of bimetallic catalysts with carbon support was carried out in this work. The pretreatment of the PtCu/C electrocatalysts may have a positive impact on their functional characteristics, including their activity and durability. For example, the ORR activity for the AC_acid material obtained after the acid treatment changed within the margin of error. At the same time, the specific activity calculated for the ESA value increased by 1.7 times for the AC_350 material obtained after the thermal treatment compared with the untreated sample.

It should be noted that the thermal treatment of the PtCu/C material at 350 °C allowed increasing its durability more than twice. The increase in durability after the thermal treatment can be attributed to several factors, including the growth of the NPs’ size and the formation of the intermetallic structure, with the latter being confirmed by the XRD results. The TXRF results indicate that after the electrochemical measurements, the heat-treated material was characterized by four times less dissolved copper than the initial sample, which also confirms the data on the high durability of the catalyst. Interestingly, the acid treatment also enhanced the durability of the PtCu/C electrocatalysts but to a lesser extent than the thermal treatment.

The sequential combination of acid and thermal pretreatment types failed to yield positive results in terms of any additional enhancement in the functional characteristics of the PtCu/C electrocatalysts. Therefore, in some instances, the pretreatment of the PtCu/C materials allows enhancing their functional characteristics that, as a result, exceed those of the commercial JM40 catalyst. This study shows the prospects of using catalyst pretreatments before their application in low-temperature fuel cells.

## Figures and Tables

**Table 1:** Some structural characteristics of the PtCu/C catalysts.

Sample	𝝎_Pt_, %	Composition (Atomic Ratio)			Average Size of NPs, nm		Crystal Lattice Parameter (a), Å
		TXRF	XRD	TXRF after CV	XRD	TEM	
AC	31	PtCu_1.26_	PtCu_0.56_	PtCu_0.25_	2.4	2.6	3.82(6)
AC_acid	35	PtCu_0.29_	PtCu_0.36_	PtCu_0.21_	2.9	2.3	3.85(2)
AC_350	31	PtCu_1.26_	PtCu_0.88_	PtCu_0.89_	7.8	4.1	3.77(7)
AC_350_acid	32	PtCu_0.80_	PtCu_0.88_	-	7.7	-	3.77(7)
AC_acid_350	35	PtCu_0.29_	PtCu_0.22_	-	20.2	-	3.86(5)

**Table 2:** The electrochemical characteristics of the studied PtCu/C catalysts.

Sample	𝝎_Pt_, %	ESA, m^2^/g_Pt_	I_k_, A/g_Pt_ at 0.9 V	I_k_, A/m^2^_Pt_ at 0.9 V	E_1/2_, V
AC	31	37	332 ± 33	9.6 ± 1.0	0.91
AC_acid	35	34	351 ± 35	10.5 ± 1.0	0.92
AC_350	31	20	308 ± 31	16.4 ± 1.6	0.91
AC_350_acid	32	25	228 ± 23	9.4 ± 0.9	0.91
AC_acid_350	35	16	142 ± 14	8.8 ± 0.9	0.89
JM40	40	59	160 ± 16	2.1 ± 0.2	0.90

**Table 3:** The electrochemical characteristics of the PtCu/C catalysts after durability testing.

Sample	𝝎_Pt_, %	ESA, m^2^/g_Pt_	I_k_, A/g_Pt_		I_k_, A/m^2^_Pt_		E_1/2_, V
			0.85 V	0.80 V	0.85 V	0.80 V	
AC	31	23	- *	207 ± 21	- *	9.2 ± 0.9	0.81
AC_acid	35	25	162 ± 16	444 ± 44	6.6 ± 0.7	18.0 ± 1.8	0.86
AC_350	31	23	352 ± 35	849 ± 85	15.0 ± 1.5	36.1 ± 3.6	0.88
JM40	40	19	138 ± 14	321 ± 32	7.1 ± 0.7	16.5 ± 1.6	0.82

*—it is impossible to determine the activity value at 0.85 V due to the high degradation of the catalyst.

## Data Availability

Not applicable.

## Supplementary Materials

The following supporting information can be downloaded at: https://www.mdpi.com/article/10.3390/ijms24032177/s1, Figure S1: The surface standardization for the AC (a), AC_acid (b), AC_350 (c), AC_350_acid (d), and AC_acid_350 (e) catalysts, where the dashed line is the first cycle and the solid line is the hundredth cycle.
