# Supplementary material for: The Effect of Pretreatment on a PtCu/C Catalyst’s Structure and Functional Characteristics
Source: Int J Mol Sci. 2023 Jan 22;24(3):2177. doi: 10.3390/ijms24032177 (PMC9916518; doi:10.3390/ijms24032177)
Supplement: Supplementary file 1 [file ijms-24-02177-s001.zip › ijms-2158787-supplementary.pdf]

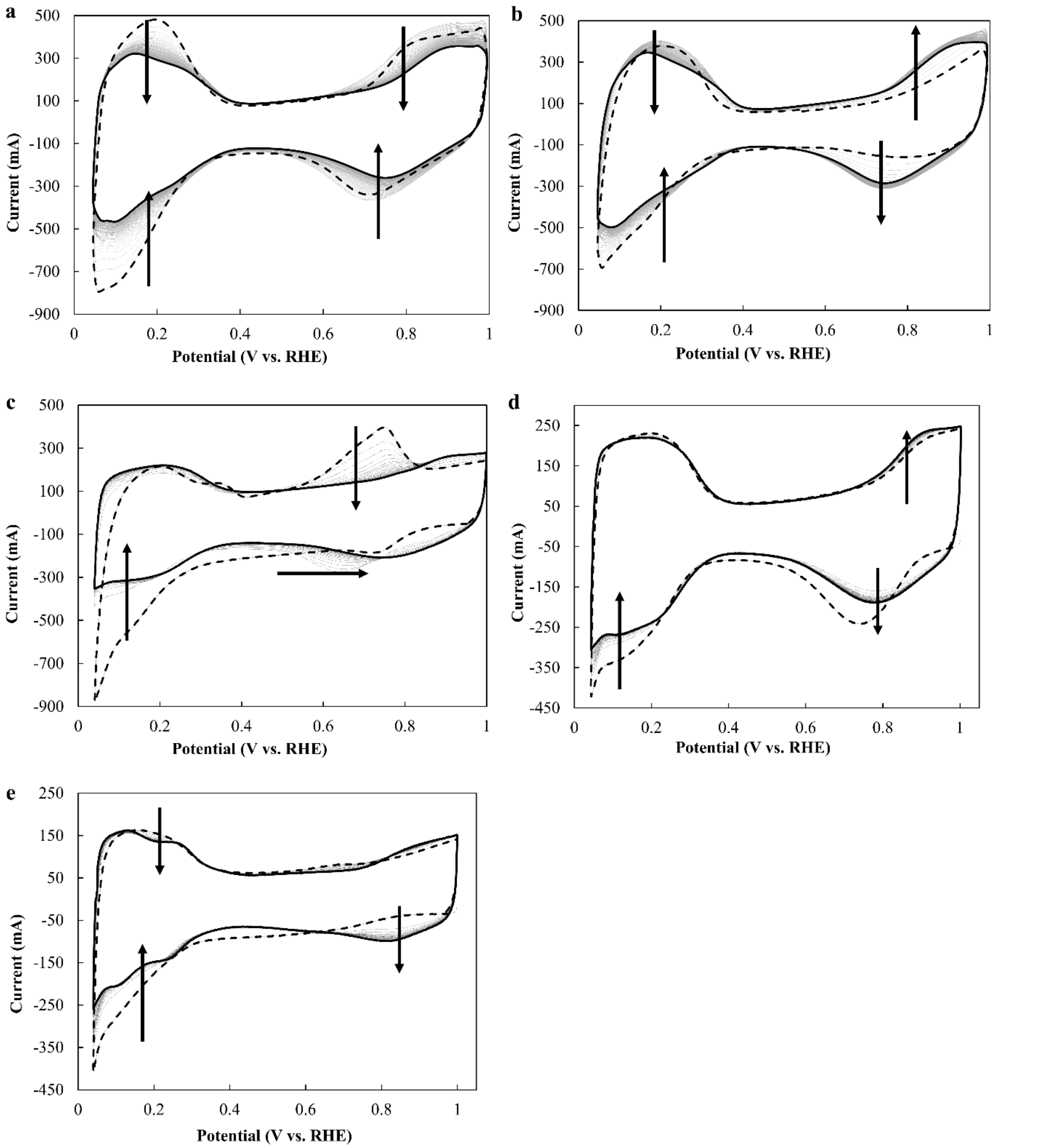

Figure S1. The surface standardization for the AC (a), AC_acid (b), AC_350 (c), AC_350_acid (d), and AC_acid_350 (e) catalysts, where the dashed line is the first cycle and the solid line is the hundredth cycle.
